# Vitamin D Deficiency after Anterior Cruciate Ligament Reconstruction Associates with Knee Osteoarthritis: A Retrospective Study

**DOI:** 10.3390/nu16173029

**Published:** 2024-09-08

**Authors:** Sonu Bae, Laura C. Schmitt, Zachary Burnett, Eric M. Milliron, Parker A. Cavendish, Robert A. Magnussen, Christopher C. Kaeding, David C. Flanigan, Tyler Barker

**Affiliations:** 1 Sports Medicine Research Institute, The Ohio State University Wexner Medical Center, Columbus, OH 43202, USA; 2 School of Health and Rehabilitation Sciences, The Ohio State University, Columbus, OH 43202, USA; 3 Department of Orthopedic Surgery, The Ohio State University Wexner Medical Center, Columbus, OH 43202, USA; 4 Department of Orthopaedics, University of Utah, Salt Lake City, UT 84108, USA

**Keywords:** vitamin D, anterior cruciate ligament, knee osteoarthritis

## Abstract

Background/Objectives: The objective of this study was to test the hypothesis that vitamin D deficiency (i.e., serum 25-hydroxyvitamin D (25(OH)D) ≤ 20 ng/mL) associates with the increased occurrence and shortened time to a knee osteoarthritis (OA) diagnosis after anterior cruciate ligament reconstruction (ACLR). Methods: This study consisted of a retrospective, case-control design. The inclusion criteria consisted of (1) patients (≥18 y) who underwent arthroscopic ACLR with (cases; *n* = 28) and without (controls; *n* = 56) a subsequent knee OA diagnosis (≥90 d from the date of ACLR) and (2) with a documented serum 25(OH)D concentration after ACLR (and before a knee OA diagnosis for the cases). Controls were matched (2:1) to cases based on sex, age at ACLR, date of ACLR, and body mass index. After matching, patients were separated into two groups: (1) vitamin D deficient (serum 25(OH)D ≤ 20 ng/mL) or (2) non-vitamin D deficient (serum 25(OH)D > 20 ng/mL). Data were extracted from the medical records. Results: Thirty-one percent (*n* = 26) of patients included were vitamin D deficient. Fifty percent (*n* = 13) of the vitamin D deficient and twenty-six percent (*n* = 15) of the non-vitamin D deficient patients were subsequently diagnosed with knee OA (*p* = 0.03). Time from ACLR to a knee OA diagnosis was significantly (*p* = 0.02) decreased in the vitamin D deficient (OA-free interval, 95% confidence interval [CI] = 7.9 to 10.9 y) compared to the non-vitamin D deficient group (OA-free interval, 95% CI = 10.5 to 12.5 y). Conclusions: Vitamin D deficiency after ACLR may serve as a prognostic biomarker for knee OA following ACLR.

## 1. Introduction

The anterior cruciate ligament (ACL) is a commonly injured and surgically repaired ligament in the knee [1]. Reported occurrence is variable, but long-term (e.g., 10- to 20-year) follow-up data indicate that up to ~90% of the individuals that sustain an ACL tear and undergo reconstruction develop knee OA [2,3,4]. Estimates indicate that ~10% of all knee OA is related to trauma, accounting for more than USD 11 billion annually in healthcare costs [5], with the prevalence expected to double by the year 2030 in the United States [6].

In the body, circulating 25-hydroxyvitamin D (25(OH)D) is a reliable indicator of vitamin D intake, storage, and status [7]. Vitamin D regulates calcium homeostasis, subchondral bone mineralization, and cartilage [8]. Low circulating 25(OH)D hinders subchondral bone and cartilage homeostasis, and is a proposed mediator of primary OA. Consistent with this logic, low or deficient serum 25(OH)D concentrations reportedly precede or accelerate the onset and progression of knee OA [9,10]. Low circulating 25(OH)D is also found with knee OA [11,12,13,14,15], but with this association, it remains unclear if low vitamin D is contributing to OA or if the underlying pathology is facilitating a circulating decrease in 25(OH)D. Nonetheless, data linking a low serum 25(OH)D concentration to the pathogenesis of OA are inconsistent [16,17,18], and the association between circulating 25(OH)D and the development of knee OA following trauma is unknown.

The association between vitamin D and outcomes following ACLR is relatively unexplored. It has been observed that there is a transient decrease in circulating 25(OH)D immediately following the acute insult of ACLR [19] and that a low circulating 25(OH)D exacerbates the strength deficits of the involved limb following ACLR [20]. More recently, primary ACL tears, revision ACLR [21], and skeletal muscle atrophy after ACLR [22] have been found to increase with low serum 25(OH)D concentrations. While these findings imply a relationship between low vitamin D and risk factors of knee OA (e.g., ACL tears and skeletal muscle atrophy and weakness), it is unknown if a low or deficient serum 25(OH)D associates with the incidence of and a shortened disease-free survival to knee OA following ACLR.

This investigation sought to evaluate the association of vitamin D deficiency with the diagnosis of knee OA after ACLR. We hypothesized that vitamin D deficiency associates with the increased occurrence of and a shortened time to a knee OA diagnosis after ACLR.

## 2. Materials and Methods

This study consisted of a retrospective, case-control design and included patients (≥18 y) who underwent arthroscopic ACLR between July 2009 and May 2018 at a single academic institution (The Ohio State University Wexner Medical Center, Columbus, OH, USA). The last day of evaluating and extracting data from the medical records was 1 June 2023, allowing a 5 y minimum of data review after ACLR for a documented diagnosis of knee OA. Patients with (cases) and without (controls) a subsequent knee OA diagnosis code in their electronic medical records after ACLR were included. Patients were further screened and included if a serum 25(OH)D concentration was recorded in their electronic medical records after ACLR. This study complies with the Declaration of Helsinki and was approved with a consent waiver by the Institutional Review Board at The Ohio State University Wexner Medical Center (Columbus, OH, USA).

Cases and controls were identified using the International Classification of Diseases (ICD) 9th and 10th Revision and Current Procedural Terminology (CPT) codes. The initial query included patients that underwent ACLR (CPT code: 29888). Case inclusion criteria subsequently consisted of those with a documented knee OA diagnosis code (ICD 9 code: 715.96 or ICD 10 code: M17) and a recorded serum 25(OH)D concentration after ACLR but before the diagnosis of knee OA documented in their medical records. Patients with a knee OA diagnosis code before or within 90 d following ACLR were excluded from the study. This study criterion identified 28 cases that underwent ACLR, were later diagnosed with knee OA, and possessed a serum 25(OH)D concentration result after ACLR but before the date of a documented knee OA diagnosis.

Control inclusion criteria consisted of patients that underwent ACLR without a documented diagnosis of knee OA but with a serum 25(OH)D concentration after surgery. Controls were matched to cases (2:1) based on sex, age at ACLR, date of ACLR, and body mass index (BMI). Investigators were blinded to serum 25(OH)D concentrations during patient identification and case-control matching. The first available serum 25(OH)D concentration after ACLR (and before knee OA diagnosis for cases) was used for this study if a patient had multiple vitamin D results after surgery. The final analysis consisted of 84 patients (cases, *n* = 28; controls, *n* = 56).

All data were extracted from electronic medical records and time aligned to the dates of ACLR and the documented diagnosis of knee OA (when applicable). Concomitant procedures performed to the meniscus (CPT codes: 29880, 29881, 29882, and 29883), articular cartilage (CPT code: 29877), other ligaments (CPT code: 27427), patella (CPT codes: 27520, 27524, and 27562), and proximal tibia (CPT code: 27530) at ACLR were identified in the electronic medical records database and documented for each participant.

Total serum 25(OH)D concentrations (sum of D_2_ and D_3_; ng/mL) were determined using a chemiluminescent immunoassay (The Ohio State University Wexner Medical Center, Columbus OH) and performed as a standard of care procedure separate from ACLR. Among other methods, individuals are commonly classified as vitamin D deficient, insufficient, or sufficient based on a serum 25(OH)D concentration ≤ 20, 21–29, or ≥30 ng/mL, respectively [23]. However, due to only a few cases with vitamin D insufficiency (*n* = 9) or sufficiency (*n* = 6) in this study, patients were subsequently separated into (1) vitamin D deficient (serum 25(OH)D ≤ 20 ng/mL) or (2) non-vitamin D deficient groups (serum 25(OH)D > 20 ng/mL). Seasons were defined as 1 March to 31 May,1 June to 31 August, 1 September to 31 November, and 1 December to 28 February for spring, summer, fall, and winter, respectively.

### Statistical Analysis

A Shapiro–Wilk test was performed prior to statistical analysis to check for normality of the data. Group (e.g., cases vs. controls or between vitamin D status groups [vitamin D deficient vs. non-vitamin D deficient]) differences were evaluated with separate *t*-tests or Mann–Whitney U tests. Statistical significance of data (e.g., group [cases and controls] and season on serum 25(OH)D concentration) was assessed with separate one-way analysis of variance (ANOVA) tests followed by a Bonferroni correction on multiple pairwise comparisons or with a Kruskal–Wallis one-way ANOVA followed by a Dwass–Steel–Critchlow–Fligner test for pairwise comparisons when appropriate. Separate Fisher’s exact or chi-square tests were performed to analyze the association between categorical variables. Knee OA disease-free survival (i.e., time from ACLR to the documented diagnosis of knee OA) was assessed with a Kaplan–Meier analysis with date of knee OA diagnosis serving as the event. Age at ACLR, BMI, season of serum 25(OH)D assessment, and concomitant procedures performed during ACLR were used as covariates when assessing the statistical significance of the time from ACLR to the documented diagnosis of knee OA. Non-normally distributed data were rank transformed prior to assessing the association between variables with a Pearson product moment linear correlation analysis. Significance was set at *p* < 0.05. All statistical analyses were performed with SYSTAT (version 13.1, Chicago, IL, USA).

## 3. Results

### 3.1. Subject Characteristics

**Figure 1:**
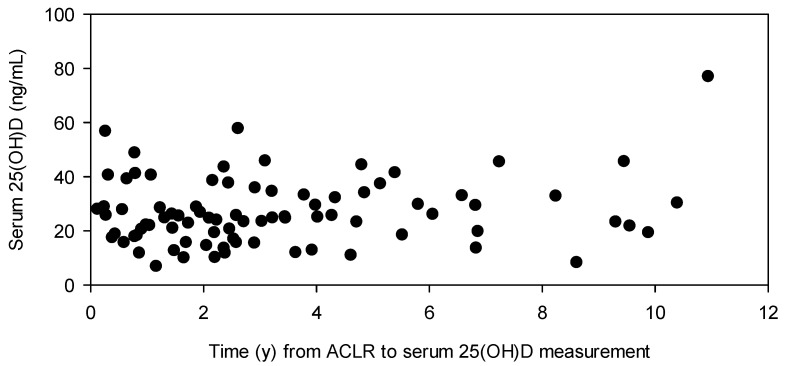
Serum 25(OH)D (ng/mL) from ACLR to measurement (y) for each patient.

This study consisted of 84 patients that underwent ACLR (ranges: age at ACLR, 19.2–58.4 y; body mass index [BMI], 19.4–66.0 kg/m^2^; serum 25(OH)D, 6.9–77.0 ng/mL), of which 67% (*n* = 56) had additional procedures at the time of surgery. Serum 25(OH)D after ACLR was not different between those with (*n* = 56; range: 6.9–57.8 ng/mL) and without (*n* = 28; range: 11.8–77.0 ng/mL) concomitant procedures performed at ACLR. Thirty-one percent (*n* = 26) of the participants possessed a serum 25(OH)D ≤ 20 ng/mL after ACLR. The median time from ACLR to serum 25(OH)D assessment was 2.50 ((IQR) 3.39) years (Figure 1).

Time from ACLR to 25(OH)D assessment and age at ACLR were not correlated with serum 25(OH)D (Table 1). BMI tended (*p* = 0.08) to inversely associate with serum 25(OH)D.

### 3.2. Cases Compared to Controls

#### 3.2.1. Patient Demographics

Sex, age at ACLR, height, body mass, and BMI were not different between cases and controls (Table 2). Approximately 68% of the cases and 66% of the controls underwent additional procedures at ACLR (Table 2 and Supplementary Table S1). The number of participants that underwent additional procedures and the types of other procedures performed at ACLR were not significantly different between the cases and controls.

#### 3.2.2. Serum 25(OH)D Concentrations

**Figure 2:**
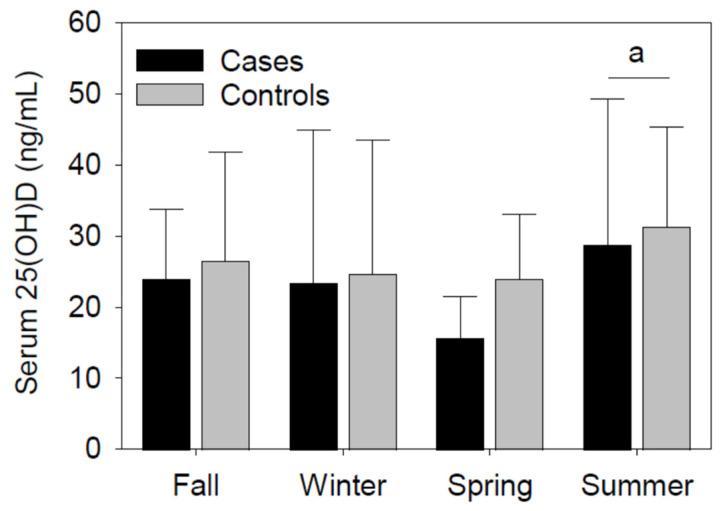
Serum 25(OH)D (ng/mL) between seasons and groups (cases vs. controls). Concentrations of serum 25(OH)D were significantly different during the summer compared to the spring. Group differences in serum 25(OH) were not significant. Fall (cases, *n* = 4; controls, *n* = 18), winter (cases, *n* = 9; controls, *n* = 17), spring (cases, *n* = 10; controls, *n* = 13), and summer (cases, *n* = 5; controls, *n* = 8) counts. Data presented as median (IQR). ^a^ *p* < 0.05 vs. spring for both cases and controls.

Time from ACLR to serum 25(OH)D measurement was not significantly different between cases and controls (Table 2). Although slightly decreased in the cases (~16%), serum 25(OH) was not significantly different between cases and controls. Consistent with the seasonal fluctuations of endogenous vitamin D levels, serum 25(OH)D was significantly (*p* < 0.05) increased in the summer compared to the spring (Figure 2). The serum 25(OH)D variations between seasons were not significantly different between the cases and controls.

#### 3.2.3. Timing of Knee OA Diagnosis for Cases and Window of Record Review for Controls

Knee OA was diagnosed in 2 cases (~7%) the first year, 3 cases (~11%) after 2 years, and 10 cases (~36%) within 5 years of ACLR. The time of data evaluation and extraction after ACLR ranged from 5.3 to 13.8 y for the controls (Table 2).

### 3.3. Vitamin D Deficient Compared to Non-Vitamin D Deficient Groups

Participants were subsequently separated into vitamin D deficient and non-vitamin D deficient groups independent of knee OA diagnosis. Sex, age at ACLR, and height were not significantly different between those with and without vitamin D deficiency (Table 3). However, in the vitamin D deficient group, body mass and BMI were significantly increased. As expected with vitamin D status demarcation, serum 25(OH)D was significantly (*p* < 0.01) decreased in the vitamin D deficient compared to the non-vitamin D deficient group. Nearly 81% of the vitamin D deficient and 60% of the non-vitamin D deficient participants underwent additional procedures at ACLR. Time from ACLR to serum 25(OH)D assessment was not significantly different between vitamin D groups. Seasons of serum 25(OH)D assessment were significantly (*p* = 0.03) different between vitamin D status groups.

**Figure 3:**
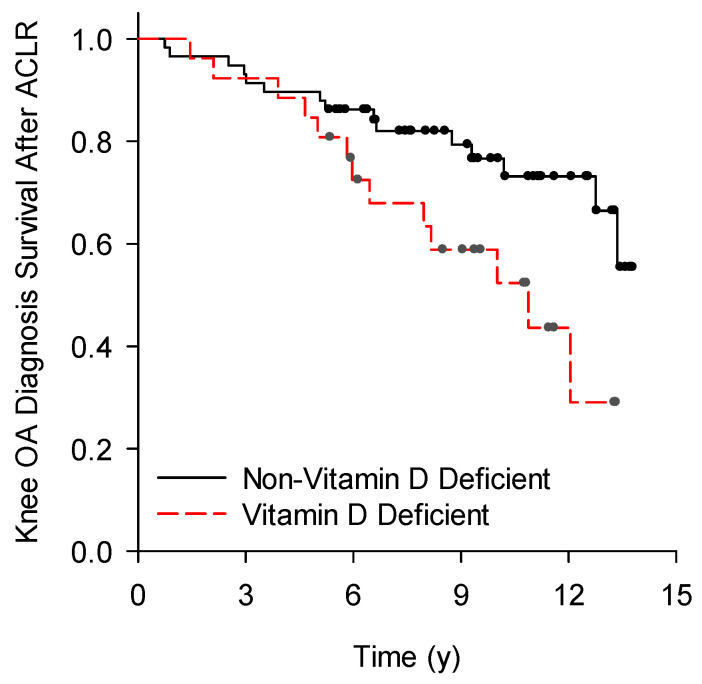
Time from ACLR to the documented diagnosis of knee OA. Time to diagnosis of knee OA was significantly (*p* = 0.02) different between vitamin D deficient and non-vitamin D deficient ACLR patients.

Fifty percent (*n* = 13) of the vitamin D deficient and twenty-six percent (*n* = 15) of the non-vitamin D deficient participants were diagnosed with knee OA after ACLR (*p* = 0.03; Table 3). Time from ACLR to the diagnosis of knee OA was significantly (*p* = 0.02) different between vitamin D deficient (OA-free interval, 95% confidence interval [CI] = 7.9 to 10.9 y) and non-vitamin D deficient groups (OA-free interval, 95% CI = 10.5 to 12.5 y; Figure 3).

## 4. Discussion

Vitamin D deficiency is associated with a variety of diseases and illnesses. This study provides original data indicating that vitamin D deficiency associates with an increased occurrence and a shortened time to a knee OA diagnosis after ACLR. Additional research examining the causative role of low serum 25(OH)D on knee OA following ACLR is needed to confirm the present findings and to extend our knowledge regarding the growing healthcare concerns of low vitamin D and knee OA.

There are limitations of this study worth discussing. This retrospective study consisted of a small sample size. However, case inclusion and exclusion criteria were strict and controls were stringently matched to cases. The pragmatic data generated from this study will be useful for establishing sample sizes for future research. It is also possible that that there could be inconsistencies with the knee OA diagnosis based on the provider and the absence of robustly defined clinical signs or symptoms implemented in the diagnosis of knee OA. Along these lines, there is a possibility that controls were lacking a knee OA diagnosis following ACLR due to receiving clinical care outside of the healthcare system utilized for data analysis. Moreover, there is a possibility that underlying health conditions or concerns in the patients of this study prompted a treating physician to order a 25(OH)D assessment. Therefore, the generalizability of the present findings could be limited. Next, calcium and parathyroid hormone (PTH) regulate endogenous vitamin D levels and contribute to the comprehensive understanding of vitamin D status. Unfortunately, calcium and PTH measurements were not routinely performed as standard of care procedures for this patient population during the window of data extraction, and therefore, results were not available. Although the time interval after ACLR in which serum 25(OH)D was assessed was not significantly different between groups, there was variability, and caution is recommended with the interpretation. Despite discrepancies in study designs, data from this investigation are comparable to those from previous research regarding the seasonal variability of serum 25(OH)D in OA, the proportion of participants (69%) with a serum 25(OH)D concentration < 30 ng/mL, and the prevalence of knee OA with a 5-year minimum evaluation phase after ACLR [18,24,25]. Future prospective research investigating the influence of supplemental vitamin D on knee OA following ACLR is encouraged to include larger sample sizes with imaging and circulating calcium and PTH results. Additionally, experimental animal research investigating the mechanisms of vitamin D on knee OA following joint trauma is also warranted.

Osteoarthritis impacts multiple tissues within an arthritic joint, including the subchondral bone. Osteoblasts are necessary for bone formation and modulated by vitamin D. Upon vitamin D receptor (VDR) binding, 1,25-dihydroxyvitamin D_3_ (1,25(OH)D)) increases osteoblast proliferation and activity, bone formation and mineralization, and angiogenesis [26,27,28]. Among others, angiogenesis is a proposed mediator in the pathophysiology of OA, and vascular endothelial growth factor (VEGF) is an angiogenic factor involved in the development and progression of OA. Vitamin D regulates VEGF expression in osteoblasts and increases angiogenesis in culture following in vivo vitamin D treatment [29]. Taken collectively, it is reasonable to infer that low serum 25(OH)D contributes to knee OA following ACLR by impairing bone mineralization and subchondral bone angiogenesis, although confirmation of this premise requires future research for later resolution, along with additional studies exploring the vitamin D and cartilage relationship.

In osteoarthritic cartilage, VDR expression increases concurrently with mediators of extracellular matrix degradation, such as matrix metalloproteinases (MMP-1, -3, and -9) [30,31,32]. The association between vitamin D and cartilage degeneration is eloquently demonstrated with in vitro data illustrating an upregulation in MMP-3 production following 1,25(OH)D stimulation [31]. While those reports and others [32] offer a unique perspective into the relationship between vitamin D and the regulation of articular cartilage degeneration at the cellular and molecular level, results linking circulating 25(OH)D to knee joint space narrowing or cartilage loss provide a contrasting narrative. For instance, one study found a low serum 25(OH)D increased cartilage loss [9], while another study found low serum 25(OH) protected against cartilage loss and osteophyte growth in knee OA [10]. Clarifying the roles of vitamin D signaling and status on knee health following ACLR is needed with further research.

A variety of risk factors associate with knee health following ACLR, including knee extensor muscle weakness [33,34,35,36]. The premise of muscular (e.g., quadriceps) weakness as a risk factor for OA underscores the necessity of preserving or improving quadriceps strength in order to protect against the deleterious events in cartilage and other localized tissues that initiate and propagate disease onset and progression [36,37]. Previous findings demonstrate impaired recovery of muscle strength of the involved limb with low circulating 25(OH)D (i.e., 25(OH)D < 30 ng/mL or hypovitaminosis D) during the rehabilitation from ACLR [20,38]. Low serum 25(OH)D also associates with other risk factors of OA, such as primary ACL tears, revision ACLR [21,39,40], and post-ACLR quadriceps atrophy [22]. Although serum 25(OH)D is readily modified by diverse interventions in OA and other conditions [41,42,43], it is unknown if increasing serum 25(OH)D attenuates muscular weakness (and other mediators of OA) and abrogates the development of knee OA following ACLR.

The regulation of endogenous vitamin D is multifactorial and includes supplemental, dietary, environmental, and genetic factors [44,45]. In knee OA, serum 25(OH)D is routinely low or decreased compared to controls [11,12,13,14]. However, opposing evidence exists [15,16], and although vitamin D deficiency associated with OA in the present investigation, serum 25(OH)D concentrations were not significantly different between cases and controls. Among others, one potential explanation for the findings here could be a lack of chronic, low-grade inflammation after ACLR and before the diagnosis of knee OA when serum 25(OH)D was assessed. In other conditions and models, inflammation or cytokines regulate the enzymatic machinery in peripheral blood cells necessary to convert 25(OH)D to 1,25(OH)_2_D_3_ [46,47,48,49] and, in the process, decrease serum 25(OH)D concentrations [19,50]. Thus, it is plausible that following the resolution of acute inflammation and cytokine deviations to ACL trauma and reconstructive surgery, serum 25(OH)D concentrations return to initial levels during the months following ACLR and before the diagnosis (or onset of signs and symptoms) of knee OA.

## 5. Conclusions

Based on the present results, we conclude that vitamin D deficiency may serve as a biomarker identifying patients at risk of developing knee OA at an accelerated rate after ACLR. Although the preliminary results here and elsewhere [20,21,22] advocate for assessing and potentially treating vitamin D deficiency after ACLR, additional research investigating the role of increasing serum 25(OH)D on the development and progression of OA and OA-related risk factors following an ACL injury and reconstruction are warranted, especially considering that the development of knee OA after ACLR is likely multifactorial.

## Figures and Tables

**Table 1:** Pearson product moment linear correlation coefficients.

	ACLR to Serum 25(OH)D		Serum 25(OH)D		Age at ACLR	
	r	*p*-Value	r	*p*-Value	r	*p*-Value
Serum 25(OH)D	0.11	0.33				
Age at ACLR	−0.13	0.24	0.16	0.16		
BMI	−0.14	0.19	−0.19	0.08	−0.05	0.69

**Table 2:** Case and control patient demographics and outcomes.

	Cases	Controls	*p*-Value
*n* (m/f)	28 (13/15)	56 (26/30)	1.00
Age at ACLR, y	34.8 (20.0)	34.3 (17.8)	0.73
Height, m	1.68 (0.13)	1.73 (0.14)	0.48
Body mass, kg	89.9 (28.7)	85.3 (25.2)	0.36
BMI, kg/m^2^	30.0 (9.4)	27.9 (7.1)	0.24
Additional procedures at ACLR, *n*			0.87
No	9	19	
Yes	19	37	
Time from ACLR to serum 25(OH)D, y	2.37 (3.54)	2.56 (3.29)	0.75
Serum 25(OH)D, ng/mL	21.6 (13.8)	25.6 (13.3)	0.14
Season of serum 25(OH) assessment, *n*			0.32
Fall	4	18	
Winter	9	17	
Spring	10	13	
Summer	5	8	
Time from ACLR to OA diagnosis, y	5.89 (5.77)	NA	NA
Time from ACLR to last records review, y	NA	9.52 (4.48)	NA

Data presented as median (interquartile range) unless otherwise noted. NA, not applicable.

**Table 3:** Vitamin D deficient compared to non-vitamin D deficient demographics and outcomes.

	Deficient	Non-Deficient	*p*-Value
*n* (m/f)	26 (11/15)	58 (28/30)	0.64
Age at ACLR, y	34.3 (19.6)	34.5 (15.5)	0.97
Height, m	1.70 (0.10)	1.72 (0.15)	0.47
Body mass, kg	97.5 (30.4)	83.5 (22.6)	0.04
BMI, kg/m^2^	32.8 (10.3)	27.5 (6.5)	0.01
Additional procedures at ACLR, *n*			0.07
No	5	23	
Yes	21	35	
Time from ACLR to serum 25(OH)D, y	2.28 (2.76)	2.66 (3.49)	0.50
Serum 25(OH)D, ng/mL	15.1 (6.1)	28.9 (13.8)	<0.01
Season of serum 25(OH)D assessment, *n*			0.03
Fall	5	17	
Winter	8	18	
Spring	12	11	
Summer	1	12	
Post-ACLR knee OA, *n*			0.03
No	13	43	
Yes	13	15	

Data presented as median (interquartile range) unless otherwise noted. Deficient, vitamin D deficient; Non-Deficient, non-vitamin D deficient.

## Data Availability

The data underlying this article will be shared upon reasonable request to the corresponding author.

## Supplementary Materials

The following supporting information can be downloaded at https://www.mdpi.com/article/10.3390/nu16173029/s1, Table S1. Other procedures (CPT codes) documented at ACLR in the cases and controls.
