# Supplementary material for: Vitamin D Deficiency after Anterior Cruciate Ligament Reconstruction Associates with Knee Osteoarthritis: A Retrospective Study
Source: Nutrients. 2024 Sep 8;16(17):3029. doi: 10.3390/nu16173029 (PMC11396950; doi:10.3390/nu16173029)
Supplement: Supplementary file 1 [file nutrients-16-03029-s001.zip › nutrients-3194444-supplementary.pdf]

Table S1. Other procedures (CPT codes) documented at ACLR in the cases and controls.

	Cases	Controls	*p*-Value
**29880, n**	4	6	0.73
**29881, n**	14	22	0.36
**29882, n**	3	12	0.37
**29883, n**	0	2	0.55
**27427, n**	1	1	1.00
**29877, n**	2	3	1.00
**29889, n**	0	2	0.55
**27530, n.**	1	0	0.33

Data presented as counts (n).
